# Effects of Fish n-3 PUFAs on Intestinal Microbiota and Immune System

**DOI:** 10.3390/md17060374

**Published:** 2019-06-22

**Authors:** Cinzia Parolini

**Affiliations:** Department of Pharmacological and Biomolecular Sciences, Università degli Studi di Milano, 20122 Milano, Italy; cinzia.parolini@unimi.it; Tel.: +39-0250318328

**Keywords:** age-related macular degeneration, gut microbiota, inflammatory bowel disease, immune system, inflammation, n-3 polyunsaturated fatty acids

## Abstract

Studies over several decades have documented the beneficial actions of n-3 polyunsaturated fatty acids (PUFAs), which are plentiful in fish oil, in different disease states. Mechanisms responsible for the efficacy of n-3 PUFAs include: (1) Reduction of triglyceride levels; (2) anti-arrhythmic and antithrombotic effects, and (3) resolution of inflammatory processes. The human microbiota project and subsequent studies using next-generation sequencing technology have highlighted that thousands of different microbial species are present in the human gut, and that there has been a significant variability of taxa in the microbiota composition among people. Several factors (gestational age, mode of delivery, diet, sanitation and antibiotic treatment) influence the bacterial community in the human gastrointestinal tract, and among these diet habits play a crucial role. The disturbances in the gut microbiota composition, i.e., gut dysbiosis, have been associated with diseases ranging from localized gastrointestinal disorders to neurologic, respiratory, metabolic, ocular, and cardiovascular illnesses. Many studies have been published about the effects of probiotics and prebiotics on the gut microbiota/microbioma. On the contrary, PUFAs in the gut microbiota have been less well defined. However, experimental studies suggested that gut microbiota, n-3 PUFAs, and host immune cells work together to ensure the intestinal wall integrity. This review discussed current evidence concerning the links among gut microbiota, n-3 PUFAs intake, and human inflammatory disease.

## 1. Introduction

Polyunsaturated fatty acids (PUFAs) are important constituents of the phospholipids of all cell membranes, where they play roles assuring the correct environment for membrane protein function, maintaining membrane fluidity, regulating cell signaling, gene expression and cellular function, and serving as substrates for the synthesis of lipid mediators. The fatty acid composition of inflammatory cells can be modified by increasing intake of marine n-3 PUFAs, which leads to a higher content of eicosapentaenoic acid (EPA) and docosahexaenoic acid (DHA) [1]. These changes in membrane phospholipid fatty acid composition might be expected to influence the function of cells involved in inflammation through: (1) Alterations of the membrane fluidity and raft structure; (2) effects on cell signalling pathways; and (3) alterations in the pattern of the lipid mediators produced [2].

Chronic inflammatory disease, such as atherosclerosis, nonalcoholic fatty liver disease (NAFLD) [3], chronic obstructive pulmonary disease (COPD) [4], inflammatory bowel disease (IBD) [5], and retinal illnesses [6], are responsible of pain, impaired function, and diminished quality of life, as well as, the associated high health-care costs and loss of productivity [7].

The human gut microbiota has become a widely discussed topic over the past decade. The intestinal microbial community lives in a mutualistic relationship with its host and is a key contributor to host metabolism. There is mounting evidence from the literature that brought to the forefront the impact of mutualistic bacterial communities of the gut microbiota on human health. Each person’s microbiome is unique, rapidly developing throughout early childhood and with differing rates of variability in adulthood. Microbial colonization runs in parallel with immune system maturation and plays a role in intestinal physiology and regulation [8]. Moreover, the network of mucosal immune cells and intestinal epithelium acts in concert to enforce barrier function, prevent mucosal infections and maintain a symbiotic relationship with the commensal flora. The disruption of this balance, whether due to genetic or environmental insults, is now recognized for having important and far-reaching impacts on immune tolerance and hypersensitivity in intestinal and extra-intestinal tissues, including the liver, the lung, the eyes, and the central nervous system [9].

Both genetic and environmental factors, including diet, geographical location, toxin/carcinogen exposure, and hormones, can influence variations in gut microbial composition [10]. The western lifestyle, including over-feeding of highly refined diets and sedentary behavior, is associated with systemic low-grade inflammation, responsible for chronic degenerative disease.

This review describes the connections among gut microbiota, n-3 PUFAs intake, and IBD or retinal disease. 

## 2. Fish n-3 PUFAs

In recent years, much focus has been placed on the beneficial effects of fish consumption strengthened by the concept that the ocean offers a wonderful resource for novel compounds. Many of the positive effects of fish, including those on dyslipidemia and heart disease, have been attributed to n-3 PUFAs, i.e., EPA and DHA. However, in addition to n-3 PUFAs, other nutrients, such as minerals, vitamins and proteins, [11] have been associated to the prevention/treatment of cardiovascular (CV) disease and associated health complications [1,12,13,14]. Indeed, in a recent experimental study, krill oil (a sustainable source of EPA and DHA) and krill proteins proved to be effective in lowering atherosclerosis development in apoE deficient mice [15]. Krill oil seems to exert this effect mainly by interfering with cholesterol synthesis, whereas krill protein may affect atherosclerosis development by supplying nitric oxide (NO) through arginine [16], thus preserving endothelial function, and, possibly, through the release of atheroprotective peptides [17].

**Figure 1:**
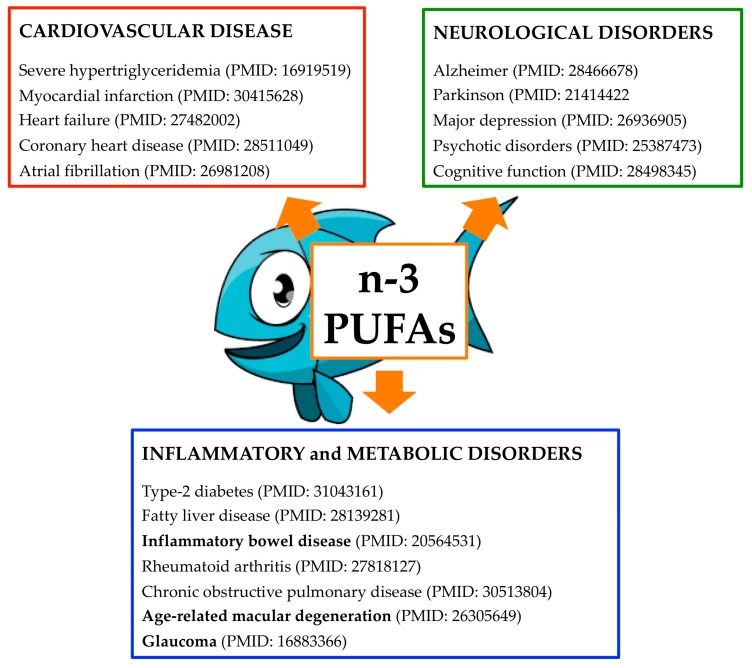
The therapeutic potential of marine n-3 polyunsaturated fatty acids (PUFAs) in human disease.

EPA and DHA are concentrated in fatty fish and marine mammals and these are the main source in the eastern diet. n-3 PUFAs are also commercially available as dietary supplements in the form of oil and capsules. In 2008, the US Food and Drug Administration (FDA) have formally stated that consumption of up to 3 g/d of marine-based n-3 PUFAs is generally regarded as safe. In line with this health claim, fish consumption is recommended in the 2015–2020 Dietary Guidelines for Americans and by the American Heart Association (Figure 1) [18].

Clinical complications of atherosclerosis like myocardial infarction, stroke and peripheral arterial disease still represent the leading cause of mortality and morbidity in the world [9,19]. Among patients who are receiving appropriate treatment with statins, the rates of CV events remain high [20,21]. In addition, recent genetic [22,23,24] and Mendelian randomization studies [25,26] have highlighted triglyceride levels as an independent risk factor for the incidence of ischemic events. Therefore, there is renewed interest in targeting triglycerides aiming at reducing the residual cardiovascular risk [21]. Lifestyle modification remains the starting point of therapy for the hypertriglyceridemia, followed by fibrates and n-3 PUFAs administration [27].

**Figure 2:**
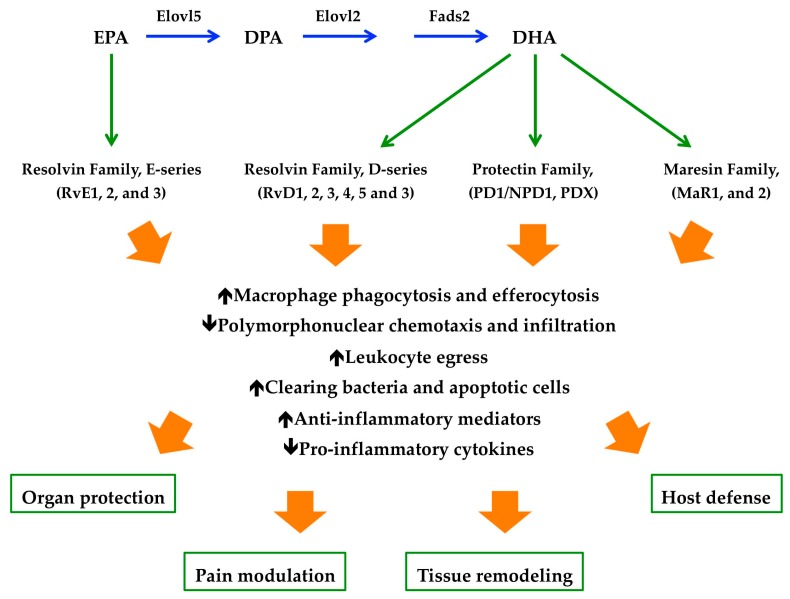
The specialized pro-resolving mediators (SPMs) biosynthetic route and physiologic actions. EPA, eicosapentaenoic acid; DPA, docosapentaenoic acid; DHA, docosahexaenoic acid; Elovl5, ELOVL fatty Acid Elongase 5; Elovl2, ELOVL fatty Acid Elongase 2; Fads2, Fatty Acid Desaturase 2.

While earlier studies on CV outcomes demonstrated favorable effects of n-3 PUFAs [28,29], subsequent clinical trials evaluating the impact of EPA (20:5n-3) and DHA (22:6n-3) administration, alone or in combination, failed to confirm the previous results [30,31,32]. These considerations led to the design of the prospective, randomized, double-blind Reduction in Cardiovascular Events with EPA-Intervention Trial (REDUCE-IT) assessing the ability of icosapent ethyl to reduce CV outcomes in high-risk statin-treated patients with triglyceride levels of at least 500 mg/dL [33,34]. In this study, the risk of both primary (25%) and secondary (26%) composite end points was significantly lower among patients treated with 2 g of icosapent ethyl than among those in the placebo group [35]. The overall rates of adverse events leading to discontinuation of trial were similar in both the clinical groups. However, serious adverse events, i.e., bleeding and the rate of hospitalization for atrial fibrillation or flutter, were observed with higher frequency among patients in the icosapent ethyl group. The authors suggested that the observed reduction in the risk of ischemic events caused by icosapent ethyl administration may be explained by metabolic effects other than a reduction of triglyceride levels, i.e., antithrombotic, membrane-stabilizing, plaque stabilizing and anti-inflammatory mechanisms [36,37,38,39]. It is known that, n-3 PUFAs, being natural agonists of GPR120 (also called free fatty acid receptor 4) [40], attenuate NF-*κ*B activation and stimulate PPARs, resulting in the reduced production of prostaglandin E2 (PGE2), TNF-α, interleukin 6 (IL-6), IL-1β, soluble E selectin, and high-sensitivity C-reactive protein [41,42]. In addition, n3-PUFAs also act as substrates for the synthesis of specialized pro-resolving mediators (SPMs), i.e., resolvins (short for resolution phase interaction products), protectins, and maresins (short for macrophage mediators in resolving inflammation) [43], reducing the inflammatory circuits of atherosclerosis [44]. These SPMs, upon activation of specific receptors, actively terminate the inflammatory reactions by increasing efferocytosis, phagocytosis and leukocytes egress (Figure 2) [45].

In line with these data, a recent study provided a link among EPA supplementation, the generation of SPM’s precursors, and their signaling pathways in the context of cardiovascular disease [46]. Specifically, the authors identified 18-HEPE as a plasma biosynthetic pathway marker of EPA supplementation, and characterized the receptor for the 18-HEPE–derived lipid mediator RvE1, i.e., ERV1/ChemR23, as a key player in atherosclerosis. The targeted deletion of Erv1/Chemr23 in 2 independent hyperlipidemic murine models was instead associated with proatherogenic signaling in macrophages, increased oxLDL uptake, reduced phagocytosis, and increased atherosclerotic plaque size and necrotic core formation. Moreover, a specific ERV1/ChemR23–expressing macrophage subtype was detected near the necrotic core into the human atherosclerotic lesions and its gene expression was increased in the plaques harvested from patients receiving statins [46,47].

The ongoing (VITAL, NCT01169259) placebo-controlled trial, designed to examine major CV events, might clarify the utility of Lovaza, a drug formulation containing ethyl ester of EPA (465 mg) and DHA (375 mg), in primary prevention of CV disease [48]. The randomized trial for evaluation in secondary prevention efficacy of combination therapy–statin and EPA (RESPECT-EPA, UMIN000012069), a study involving statin-treated patients in Japan, and the effect of Vascepa (a preparation of EPA as ethyl ester) on improving coronary atherosclerosis in people with high triglyceride levels taking statin therapy trial (EVAPORATE, NCT02926027), which is examining changes in coronary plaque over 9 to 18 months, will provide further information on the effects of these agents. In addition, the statin residual risk reduction with Epanova in hiGh cardiovascular risk patients with hypertriglyceridemia (STRENGTH NCT02104817) is an ongoing trial that will be exploring residual cardiovascular risk reduction with 4 g daily of EPA/DHA (Epanova) versus corn oil in patients on statin therapy.

Finally, it is should be noted that n-3 PUFAs have dramatically modified the biochemical composition of the lipid rafts, i.e., the dynamic sphingolipid-cholesterol enriched regions of the membrane that concentrate specific signaling proteins [49]. These lipid microdomanins play a crucial role in the CD4+ T cell activation and differentiation, and downstream in the B cell activity [50]. Several in vitro and in vivo data have shown that n-3 PUFAs displaced many of the signaling proteins necessary for CD4+ T cell activation [51]. In addition, n-3 PUFAs suppressed CD4+T cell polarization into Th1 and Th17 cells, probably by altering the IL-6/gp130/STAT3 pathway [52]. On the other hand, n-3 PUFAs or SPMs boosted humoral immunity by influencing B cell development in the bone marrow, B cell activation and antibody production in response to antigen [53]. Although these data need to be confirmed and the underlying mechanisms better clarified, it can be speculated that n-3 PUFAs may contribute in the attenuation of autoimmune and chronic inflammatory disease [51].

## 3. The Gut Microbiota

The gastrointestinal (GI) tract is a passage formed by mucosa, submucosa, and smooth muscle containing a huge network of nerves and blood vessels. The mucosa is responsible for: Digestion and absorption of nutrients from the diet; protection of the body against physical and chemical damage from luminal contents; and supply of immunity [54,55]. The small intestinal epithelium, the single cell layer that forms the luminal surface, is folded to form villi and crypts. Both these structures serve to increase the total absorption surface of the intestine. Moreover, in the mucosa different types of cells are present, such as enterocytes (the main cellular component accounting for about 80%), Paneth, goblet, and enteroendocrine (comprise just 1% of the epithelium) [56]. Enteroendocrine cells, distributed throughout the GI tract, form a large endocrine organ that control: (1) GI secretion and motility; (2) regulation of food intake; (3) postprandial glucose levels; and (4) direct communication with neurons innervating the GI tract (these cells are part of the gut brain axis) [57].

The mammalian intestine is colonized by a complex community of microorganisms, called microbiota, constituted by bacteria (approximately 4 × 10^13^), archaea, viruses (especially bacteriophages), protozoans and fungi [58]. Shotgun metagenomic sequencing through random sequencing of all genes established that the human gut possesses a bacterial microbiome that is predominated by phyla, such as *Firmicutes*, *Bacteroidetes*, *Actinobacteria*, *Proteobacteria*, *Verrucomicrobia*. Firmicutes and Bacteroidetes represent 90% of the gut microbiota [9]. It is important to remember that the colonization of the human gut starts prenatally and continues after birth, reaching the adult microbiota state between the ages of 2 and 5 years [59]. Following birth, diverse microbes colonize the human intestine, and several factors are known to affect this process, i.e., gestational age, mode of delivery, diet (breast milk vs formula), sanitation and antibiotic treatment [60]. Several studies showed a positive correlation between human milk oligosaccharides and the number of *Bifidobacterium* demonstrating that milk oligosaccharides have a probiotic effect by stimulating the development of *Bifidobacterium*-rich microbiota [61]. Furthermore, in addition to proteins, fats, carbohydrates and endocannabinoids, human breast milk contains immunoglobulins (IgA and IgG), antimicrobial compounds (lysozyme and lactoferrin), immune-regulatory cytokines (e.g. TGF-β and IL-10), and lymphocytes that express gut homing markers [62,63]. Altogether these data indicate that intestinal bacterial colonization dramatically influence the development of the host immune system in early life and affects health and disease in later life, as indicated by the loss of immune function in germ-free (GF) mice [63,64,65].

A significant change in the gut microbiota composition accompanies the weaning from the mother and the introduction of solid foods, with enhanced colonization of butyrate producers, including *Bacteroides* and certain *Clostridium* species [66]. Once established, the composition of the gut microbiota is relatively stable throughout adult life in the absence of perturbations such as long-term dietary changes, disease-associated dysbiosis, or the use of antibiotics. However, centenarian’s microbiota showed decreased levels of *Bacteroides*, *Bifidobacterium* and Enterobacteriaceae, while *Clostridium* species levels were increased compared with younger adults. For these reasons, dietary-intervention studies are warranted to investigate whether changing the dietary pattern of elderly individuals can alter their gut microbiota in a way that is beneficial to their general health [67].

### The Gut Microbiota and the Immune System

A noticeable example of the symbiotic effects of the microbiome is the immune system, whose normal development and behavior are strongly influenced by microbial metabolites that are produced by: (i) Bacteria from dietary components; (ii) the host and biochemically modified by gut bacteria; and (iii) by gut microbes. In this context, a complex interplay between the local microbiota, the intestinal epithelium and the resident immune cells has begun to emerge, in which all participants actively foster GI homeostasis [9]. Segmented filamentous bacteria are best known for their ability to induce the differentiation of naïve T cells to form antigen-specific Th17 cells in the ileum of mice [68] and humans [69]. Th17 cells play a critical role in host defense against extracellular pathogens (i.e., fungi and extracellular bacteria [70]) and tissue homeostasis (i.e., promoting epithelial barrier functions [71]) but can induce autoimmunity [72]. The mechanisms implicated in the balance between ‘pathogenic’ and ‘non-pathogenic’ Th17 cell states are still unknown. However, treatment of non-pathogenic Th17 cells with IL-23 converts them into a pathogenic phenotype, suggesting that IL-23 is a cytokine that drives the functional phenotype of Th17 cells [73]. Furthermore, the metabolic relationship between diets and immune cells in the gut has been strengthened by the finding that short chain fatty acids (SCFAs) promoted B cell metabolism [74] and the development and function of colonic regulatory T (Treg) cells [75]. Treg cells have played a role of gatekeeper of commensal tolerance by the immune system, through suppression of aberrant T cell responses. A balance between Th17 and Treg cell differentiation has been demonstrated [76]. Beyond SCFAs, gut microbiota have produced several additional immunologically important metabolites from food components. Dietary tryptophan, for instance, is processed to different indole derivatives, which can act as ligands for the aryl hydrocarbon receptor (AhR) [77], a ligand-activated transcription factor which plays a crucial role in the development of multiple tissues. In the intestinal mucosa, AhR is required for postnatal maintenance of Group 3 innate lymphoid cells (ILC3s) producing IL-22, as well as for the formation of isolated lymphoid follicles [9]. IL-22 is a cytokine that supports the integrity of the intestinal mucosa by inducing the secretion of antimicrobial peptides from epithelial cells, the production of mucins and the proliferation of intestinal goblet cells [78]. Highly reactive polyamines, i.e., putrescine, spermidine and spermine, derive from arginine by the action of gut microbiota, are involved in the development and maintenance of the intestinal mucosa and resident immune cells [79]. In addition, arginine itself is an important modulator of the immunometabolism of macrophages and T cells, and thus affects their effector functions [80]. Moreover, free taurine generated by deconjugation of primary bile acids [81] can promote the activation of the NLRP6 inflammasome and the production IL-18, supporting epithelial barrier function and maintenance [82]. In addition, bile acids are able to downregulate the expression of pro-inflammatory cytokines from monocytes, macrophages, dendritic cells and Kupffer cells [9].

On the other hand, a deficit of vitamin B1 is responsible for the reduction in the number of naïve B cells in Peyer’s patches, due to their dependence on this Krebs cycle cofactor, but has a neutral effect on IgA+ plasma cells present in the lamina propria [83]. 

## 4. Human Disease, n-3 PUFAs and Gut Microbiota

**Figure 3:**
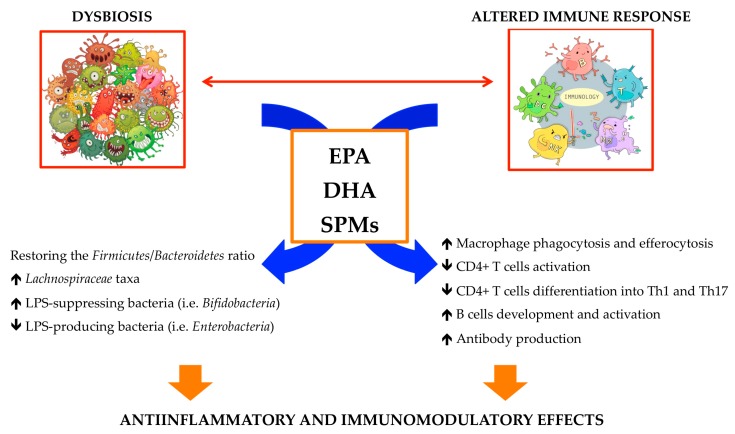
n-3 PUFAs and SPMs interplay with gut microbiota and the immune system.

Inflammation seems to be the common denominator among the above described seemingly unrelated biological entities, i.e., the gut microbiome, the immune system, and n-3 PUFAs. Inflammation is currently accepted to play a key role in the progression of several chronic diseases, such as atherosclerosis, inflammatory bowel disease, cancer, diabetes, neurodegenerative syndromes, etc. [9]. In addition, as above described several evidences support the role of both the microbiota and the n-3 PUFAs in regulating inflammation and the immune system [45,52,74]. Moreover, dietary n-3 PUFAs, affecting gut integrity, have been shown to reduce clinical colitis and colonic immunopathology by improving epithelial barrier function in animal models [84]. Indeed, in clinical studies n-3 PUFAs have demonstrated the ability of: (i) Decreasing the *Firmicutes*/*Bacteroidetes* ratio; (ii) decreasing the levels of *Coprococcus* and *Facecalibacterium*; (iii) increasing the abundance of butyrate-producing bacterial genera, i.e., *Bifidobacterium*, *Lachnospira*, *Roseburia* and *Lactobacillus* [85,86,87]. These data were in line with those obtained in a subsequent study where the authors also found a significant correlation between n-3 PUFAs plasma levels and SCFA-producing bacteria, i.e., *Lachnospiraceae* family [85]. In addition, a diet supplemented with n-3 PUFAs was able to prevent neuropsychiatric disorders and dysbiosis induced by social instability stress during adolescence, and these effects were maintained through adulthood [88,89] supporting the concept that a healthy diet may have long-lasting beneficial effects and help fight off neurodegenerative diseases. Altogether these data allow hypothesizing a link among n-3 PUFAs intake, gut microbiome shaping and immune system modulation with the final common aim of hampering inflammatory-based disease (Figure 3). Accordingly, this review will particularly focus on the recent studies regarding the therapeutic potential of the combination between fish n-3 PUFAs and probiotic/prebiotic in the IBD and retinal disease.

### 4.1. Inflammatory Bowel Disease

**Figure 4:**
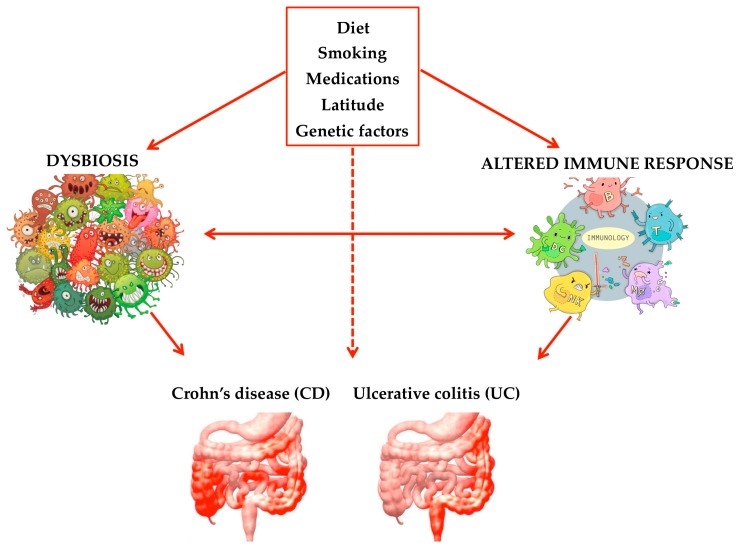
The pathogenic factors involved in the development of Crohn’s disease (CD) and ulcerative colitis (UC).

IBD, specifically Crohn’s disease (CD) and ulcerative colitis (UC), are relapsing and remitting inflammatory diseases of the GI tract without a clear etiology. The symptoms include abdominal pain, diarrhea, weight loss, ulceration, perforation, and bowel obstruction. Although the all picture of the pathogenesis of IBD remains unclear, aberrations in genetics, imbalances in the gut microbiome, dietary and lifestyle factors such as cigarette smoking, medications, and environmental triggers (i.e., geographical location and social status) are all believed to play a role in the disease’s development (Figure 4).

Genome-wide association studies (GWAS) have identified 242 loci associated to the presence of IBD [5,90]. For example, variants at the NOD2 and CDH1 loci confer the largest increase in relative risk of IBD and have been associated to UC, respectively [91,92,93]. The NOD2 gene encodes a protein that is activated within the cytoplasm of macrophages and dendritic cells by bacterial ligands [94], whereas CDH1 gene has a critical role in cell-cell adhesion and is also required for epithelial cell tight junction formation [95]. Moreover, a meta-analysis revealed that genetic variations in TLR4 gene, the receptor for LPS, conferred a statistically significant risk of developing CD and UC [96,97]. In addition, polymorphisms in TLR2, the main receptor for gram-positive bacteria, have been associated with IBD in humans and there is an inflammation-dependent induction of TLR2 expression in intestinal macrophages [98]. 

The hyper-responsiveness of T cells toward non-pathogenic antigens could represent one of the possible etiologies for IBD. The presence of antibodies against commensal microbial antigens and autoantigens, such as anti-*Saccharomyces cerevisiae*, and anti-*Pseudomonas fluorescens*-associated sequence 12 [99,100,101], has been associated to dysbiosis and loss of microbiota responsible for the gut mucus barrier integrity. Since it has been shown that colitis-prone genetically predisposed GF mice colonized by IBD-associated-microbiota developed severe colitis compared to those that were colonized by healthy human microbiota, it can be hypothesized that gut dysbiosis contributes to IBD pathogenesis [102]. Together, these findings strongly indicate a bidirectional relationship between such diseases and gut dysbiosis, in which dysbiosis potentially contributes to the onset of IBD and also serves as a secondary consequence of gut inflammation [103,104].

It is well known that the microbiota and the gut have a symbiotic relationship: The human host supplies the nutrients needed for the survival of the microbes, and these latter protect the host against pathogens, and act as regulatory factors of the immune responses [56]. An aberrant mucosal immune system and irregular mucosal epithelium with increased intestinal permeability (IP) may permit the translocation of bacterial-derived toxins causing gut inflammation. The communication between the gut microbiota system and all the organs of the human body is regulated by the IP [105,106]. Indeed, IP degree is very changeable and results from the interconnection between several factors: Type of diet; gene expression; intestinal/liver pathology; surface mucus; integrity of tight junctions; and production of immunoglobulins [107]. Several investigators detected a decrease of several protective bacteria (i.e., *Fecalibacterium prausnitzii*, *Clostridium* clusters IV and XIVa, some *Bacteroides* species, *Bifidobacterium*) and an increase of harmful bacteria (i.e., adherent-invasive *Escherichia coli*, *Fusobacterium*, *Campylobacter concisus*, *Enterohepatic Helicobacter*, *Clostridium difficile*, *Veillonella*) in IBD patients [108,109].

Several mechanisms are responsible for the intestinal inflammation caused by the consumption of high-fat diets (HFDs), including both changes in the intestinal barrier and composition of the intestinal microbiota. Experimental and clinical data indicated a direct correlation between plasma endotoxin levels and dietary fat intake, which suggested an increase of the IP [110,111] due to the decrease of epithelial tight junction proteins, such as occludin. Indeed, several experimental studies showed a dramatic decrease of intestinal occludin expression associated to the administration of a HFD [112,113,114]. On the other hand, high consumption of carbohydrates, such as glucose, sucrose, lactose, or fructose, overwhelms absorptive mechanisms of the intestine, resulting in high luminal sugar concentrations used by the microbiota as an energy source [115]. In support of this hypothesis, consumption of a high sugar diet was demonstrated to promote intestinal dysbiosis, the expansion of harmful bacteria (such as, *Ruminococcus torques*, *Bacteroides*, *Prevotella*), increased IP and inflammation [116,117]. In contrast, a fish-oil based diet has been associated with *Lactobacillus* and *Akkermansia muciniphila* blooming, and reduced gut inflammation [118,119]. The observed increase in the abundance of *Akkermansia muciniphila* could be a paradox because this is a mucin-user bacterium and at the same time, it is crucial for the maintenance of the mucus layer integrity [120]. However, this latest effect appeared to be more significant as proved by the fact that increased intestinal levels of *Akkermansia muciniphila* has been associated to a mucus layer thickening and a reduction of IP [121]. 

Recently, the treatment for IBD has made progress from simply controlling symptoms to modifying the course of the disease by achieving and maintaining remission which is defined as complete mucosal healing and normalization of blood markers, as well as disappearance of symptoms [122]. However, the development of a safer and more effective novel treatment for IBD is in great need.

The role of gut microbiota in colitis development was confirmed by using animal models. GF mice displayed minimal inflammation or delayed onset of chemically and genetically induced colitis compared to the conventionally raised (CONV-R) animals [123,124,125]. However, higher mortality was seen in GF than CONV-R mice after giving dextran sulfate sodium (DSS), due to massive gut epithelial injury [126]. The seemingly paradoxical phenomenon could be explained by the lack of immune maturation and/or tolerance as well as the impairment of epithelial turnover (which is dependent on commensal colonization) in GF mouse intestine [127,128]. Gut microbiota metabolites act as important signals for the monitoring of the correct function of the epithelial barrier and the immune cells. Similarly, immune-driven signals central to gut homeostasis can also modulate the metabolism of immune cells [129]. It is well known that several hematopoietic cells produce IL-10 and its importance for maintaining tolerance within the intestinal microbiota come from experimental observations using IL-10- or IL-10R-deficient mice (both these mouse models develop spontaneous colitis) (see below, [130]), and clinical data (IBD patients are characterized by decreased levels of the anti-inflammatory cytokine IL-10). In line with these results, administration of genetically modified probiotic, i.e., *Lactococcus lactis* expressing IL-10, had demonstrated a significant remission of disease activity in CD patients. Furthermore, oligofructose-enriched inulin (OF-IN) administration was able to induce an improvement in CD associated to a reduction of the *Ruminococcus gnavus*. In addition, it has been recorded that the intake of this prebiotic is able to increase the abundance of the *Bifidobacterium longum*. This bacteria neutralizes reactive oxygen species and exerts anti-inflammatory effects at the site of inflammation, reducing gastrointestinal discomfort and tissue injury [131]. Previous studies also showed that different probiotic combinations, especially *Bifidobacterium longum* or a multistrain mix of *Bifidobacterium longum*, *Lactobacillus acidophilus*, and *Streptococcus faecalis*, increased expression of tight junction proteins in IBD [132]. Furthermore, VSL#3^®^, a mixture of 4 species of Lactobacillus, 3 species of Bifidobacterium, and 1 species of Streptococcus, has been shown to: (i) Improve epithelial barrier damage; (ii) induce remission in active UC; and (iii) decrease pro-inflammatory mucosal cytokine expression [133].

Meta-analyses revealed a low incidence of IBD in eskimos, whose diet is particularly rich in n-3 PUFAs [134]. Starting from these clinical evidences, the impact of dietary n-3 PUFAs has been evaluated in different models of colitis. All the results obtained in these experiments are coherent and indicate that n-3 PUFAs decrease chemically-induced intestinal tissue damage and inflammation [135]. Similarly, in fat-1 transgenic mice, characterized by high levels of endogenous n-3 PUFAs, the intestinal tissue damage was significantly reduced compared to wild type mice, together with decreased expression of TNF-α, IL-1β, and increased synthesis of SPMs [136,137]. In addition, the fat-1 mice displayed higher gut microbiota diversity and more abundance of Verrucomicrobiota plylum (*Akkermansia* genus) compared to wild-type [138].

Moreover, a diet high in fibers and n-3 PUFAs is protective for the development of IBD, whereas a diet high in refined sugars, complex carbohydrates, and n-6 PUFAs (i.e., red meat), increases the risk of acquiring IBD. A number of studies indicated that homeostasis is crucial to have a good n-3 to n-6 PUFAs ratio, the former being anti-inflammatory and the latter pro-inflammatory molecules [1,139]. Therefore, dietary n-3 PUFAs supplementation is encouraged as anti-inflammatory adjuvants for IBD [140,141]. On the contrary, a low-fiber diet may increase the risk of CD, and, if associated to high consumption of sugar and soft drinks, it may also increase UC risk [142]. However, thus far, the effects of dietary interventions on CD and UC are uncertain [143]. The cornerstone is that dietary fiber is a plant-based carbohydrate that resists digestion by intestinal and pancreatic enzymes in the human GI tract. Soluble and insoluble dietary fibers are essential for gastrointestinal mucosa health because they serve as important substrates for the gut microbiota. The fermentation products selectively promote the growth of beneficial Bifidobacteria and Lactobacilli and exert anti-inflammatory (such as, inhibition of NFκB transcription) and anti-carcinogenic functions [144].

Two large clinical trials designed at evaluating the effects of n-3 PUFA supplementation on CD provided conflicting results and concluded that supplementation was not effective in preventing CD relapse [145,146]. Intriguingly, another study suggested that IBD patients that achieved a n-3/n-6 ratio of 1 maintained disease remission at a significantly higher rate compared to those did not reach this goal [147]. A double-blind, randomized study was carried out in 38 pediatric CD patients, who received, for 12 months, 5-aminosalicylic acid (50 mg/kg/d) + n-3 PUFAs (3 capsule/d, each capsule contained 400 mg/g EPA and 200 mg/g DHA) or 5-5-aminosalicylic acid (50 mg/kg/d) + olive oil placebo capsules [148]. The results indicated that the addition of enteric-coated capsules to a conventional therapy with 5-aminosalicylic acid delayed the relapse of the disease even though it could not prevent it. Another study used enteric-coated n-3 PUFAs capsules for remission maintenance in adult CD patients at high risk of relapse, and found n-3 PUFAs to be more effective than placebo [149]. However, two multicentre, randomized, double-blind, placebo-controlled studies, called Epanova Program in Crohn’s 1 and 2, (EPIC1 and EPIC2), found that relapse occurred in 32% with n-3 PUFAs and 36% with placebo in EPIC1, and 48% with n-3 PUFAs and 49% with placebo in EPIC2, indicating that n-3 PUFAs did not reduce the rate of relapse in patients with quiescent CD [146]. Of interest, two random double-blind placebo crossover studies reported significant improvement of the disease [150] and of the oxidative stress status in active UC patients receiving sulfasalazine, respectively [151]. Thus, the efficacy of n-3 PUFA or fish oil against CD or UC is, at best, marginal. However, none of the studies reported any adverse effects associated with n-3 PUFA supplementation. A recent meta-analysis of observational studies showed that fish consumption was inversely associated with the risk of CD. Moreover, there was a strong inverse association between dietary n-3 PUFAs intake and the risk of UC [152]. Previously published studies revealed that fish consumption and dietary n-3 PUFAs intake might play a role in the etiology of IBD [153,154]. In line with these findings, one study revealed that the Mediterranean diet, rich in fish and seafood, reduced inflammation [151] and normalized the gut microbiome in CD patients, i.e., an increase in Bacteroidetes and *Clostridium* clusters, and a reduction of Protebacteria and Bacillaceae [155]. In a pilot study, short-term EPA-supplementation reduced mucosal inflammation favoring an improvement of both endoscopic and histological inflammation in almost all patients, together with a significant up-regulation of IL-10 expression, and a reduction of STAT3 activation [156]. Moreover, microbiota analysis showed that EPA treatment increased the family Porphyromonadaceae and the genus *Parabacteroides*, and reduced the genus *Bacteroides* that include mucolytic species [157].

In addition to fats and fibers, bioactive amino acids and oligopeptides are derived from food proteins through chemical and enzymatic hydrolysis, or bacterial metabolism [158,159]. In vitro and in vivo models of IBD have been used to test the antioxidant properties of amino acids. Cysteine, methionine, taurine, tryptophan, tyrosine, phenylalanine are able to reduce the oxidative stress, because of the presence of a thiol group and of an aromatic ring [142,160]. In addition, dietary cysteine was effective at reducing the intestinal inflammatory responses and at enhancing mucin synthesis in DSS-induced colitis animal models [161,162,163]. The anti-inflammatory activity of these bioactive molecules involves the activation of the calcium-sensing receptor (CaSR), a protein controlling different cellular activities (i.e., proliferation, differentiation, and apoptosis) [164,165]. On the other hand, mucins are cysteine-rich glycoproteins, secreted by goblet cells, and very important for intestinal epithelial integrity. 

### 4.2. Retinal Disease

Despite major advances in treatment and management, retinal disease cause over three-quarters of all cases of irreversible vision loss in the western world. Recent estimates indicated that the leading causes of irreversible severe visual impairment in those >50 years of age were diabetic retinopathy, age-related macular degeneration (AMD) and glaucoma, affecting~5.4 million people globally [166]. Retinal disease manifest abnormal angiogenesis, proliferative neovascularization, excessive vascular permeability, immunoregulatory dysfunction, alterations in physiologic reduction-oxidation (redox) balance, or retinal pigment epithelial (RPE) cell degeneration. 

Pathological factors affecting the retina health include ischemia, light exposure, oxidative stress, apoptosis, inflammation, neuroactive cell signaling molecules, and aging. Moreover, plasma levels of high-sensitivity C-reactive protein and pro-inflammatory cytokines, such as TNF-α and IL-6, have been elevated in subjects with essential hypertension, coronary heart disease, type 2 diabetes, retinal disease, and inflammatory bowel disease [167,168,169,170]. Anti-inflammatory therapy with canakinumab, targeting the IL-1β innate immunity pathway, markedly reduced the plasma levels of IL-6 and high-sensitivity C-reactive protein [171], and led to a significantly lower rate of recurrent cardiovascular events than placebo, independent of lipid-level lowering [172]. These studies provide the proof of concept that the removal of pro-inflammatory markers reduces the inflammatory-based disease risk.

Within the neural retina, phosphatidylcholine (PC) and phosphatidylethanolamine (PEA) represent the predominant PUFA-rich lipid class, followed by phosphatidylserine (PS) and phosphatidylinositol (PI). Arachidonic acid (AA) is the major PUFA in the neural and vascular tissue of the retina [173,174]. PUFA-containing phospholipids enter the RPE or photoreceptor inner segment via a receptor mediated transport process. Tracer studies have indicated that DHA-containing phospholipids are then integrated as structural constituents of photoreceptor disk membranes and are retained in proximity to rhodopsin molecules across the life span of these organelles [175]. Several bioactive molecules play a crucial role in the retinal disease, i.e., eicosanoids, angiogenic factors, matrix metalloproteinases (MMPs), reactive oxygen species, cyclic nucleotides, neurotransmitters and neuromodulators, proinflammatory and immunoregulatory cytokines, and inflammatory phospholipids. It has been shown that n-3 PUFAs are able to modulate the production, activation, and potency of these molecules. DHA and its substrate, EPA, influence eicosanoid metabolism by reducing n-6 PUFAs levels (mainly arachidonic acid, AA, 20:4n-6) and compete for enzymes (cyclooxygenase, COX and lipoxygenase, LOX) producing AA-based angiogenic and proinflammatory eicosanoids [176]. 

As above mentioned, intestinal bacteria are often considered a hidden metabolic organ, since they play a significant role in human nutrition and metabolism. It has been demonstrated that CONV-R mice had reduced levels of multiple PC species in lens and retina compared to GF animals, which suggested that the gut microbiota influenced the lens and retinal lipid composition [177]. Antibiotics depletion of gut microbiota in a spontaneous mouse model of uveitis resulted in significant attenuation of the retinal disease, associated with a reduced population of Th17 cells in the intestinal lamina propria (LP). Co-housing GF with CON-R mice restored disease development [178]. Recently, an experimental study demonstrated subclinical intestinal dysfunction in retinal disease leading to enhanced migration of leukocytes between the intestine and the eye. These alterations were improved by 3-week pre-treatment of SCFA sthrough induction of Treg in several tissues and inhibition of T cell differentiation into Th1 and Th17 [179]. Altogether, these data suggest that commensal microbiota may serve as triggers of immune responses and offer clues to the environmental origin of ocular inflammation [180]. 

#### 4.2.1. Age-Related Macular Degeneration

**Figure 5:**
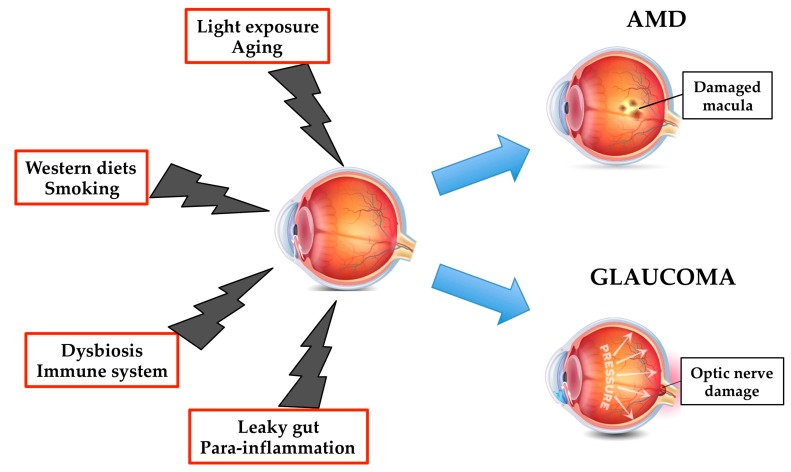
Altered dietary habits, dysbiosis and leaky gut, and low grade inflammation, together with aging, smoking and light exposure may influence the risk and progression of age-related macular degeneration (AMD) and glaucoma.

In patients older than 55 years, age-related macular degeneration (AMD) is considered the leading cause of serious visual loss in the developed world [97,181], and the third leading cause of blindness in the world [182]. AMD is a degenerative disease caused by the concomitant presence of non-modifiable (genetics, sex, age) and modifiable factors (nutritional status, smoking status) (Figure 5) [183]. Several genetic associations have been found, the most studied are those related to inflammatory genes such as complement factor H and certain complement components (i.e., C3 and C2) [184]. Recently, a tissue-adaptive response, called para-inflammation, where the innate immune system engages a low-grade inflammatory response to restore tissue homeostasis, has been associated to the pathogenesis of ADM [185].

The development of treatments targeting vascular endothelial growth factor (VEGF) over the past 10 years has seen a dramatic reduction in vision loss from advanced AMD. However, with long-term treatment, many patients with AMD continue to lose vision [186]

Experimental and epidemiologic studies have indicated a link between the dietary consumption of xanthophyll carotenoids, i.e., lutein (L) and zeaxanthin (Z), or n-3 PUFAs and the reduced risk of advanced AMD [187,188,189,190,191]. Specifically, they found a direct correlation between the daily intake of these xanthophyll carotenoids and their concentrations in both plasma and macula, the latter being the district were they used to synthetize the macular pigment [192,193,194,195]. This is an important event, because the macular pigments exhibit protective functions against oxidative stress and inflammation, both factors implicated in the pathogenesis of AMD [196].

On the other hand, the neuroprotective role of n-3 PUFAs has been proved by several epidemiological studies that observed a decreased risk of ADM in subjects with high intake of n-3 PUFAs [173,197]. In a large prospective study, with 24–28 years of follow-up, n-3 PUFAs and fatty fish intakes were associated to a lower risk of visually intermediate AMD, but no relationship was found with the risk of advanced AMD [198]. Moreover, in healthy volunteers, macular pigment density was associated not only with L and Z plasma concentrations, but also with plasma levels of phospholipids containing n-3 PUFAs, particularly EPA and docosapentaenoic acid (DPA) [199]. The latter being the second most abundant n-3 PUFAs found within the retina and a metabolic intermediary between EPA and DHA [200]. In a mouse model of AMD, the administration of L, Z and n-3 PUFAs caused retinal lesion regression associated with a reduced expression of pathologic genes, and preservation of photoreceptors [201]. Moreover, in another experimental model of AMD, the supplementation with n-3 PUFAs reduced the photoreceptor damage, and the mRNA and protein expression of inflammatory mediators [202,203,204]. Altogether these data indicated that the efficacy of n-3 PUFAs could be explained by a combination between the resolution of inflammation and the induction of a regenerative process. Coherent with this hypothesis is the discovery of elovanoids, bioactive lipid mediators derived from very long chain (VLC) n-3 PUFAs by the action of the elongase ELOVL4. Indeed, the contemporary presence of free VLC n-3 PUFAs and oxidative stress seems to generate a pro-homeostatic and cytoprotective milieu [205]. In line with these results, it has been observed that mutant EVOVL4 caused juvenile macular degeneration due to protein mislocalization and photoreceptor cell death [205]. Recently, a linear association between the dose of fish consumption and the risk of AMD was found, strengthening the role of n-3 PUFAs in AMD pathogenesis [206]. Taken together, these results suggested that the metabolic pathway responsible for the synthesis of the elovanoids should be the object of future studies because it could open a new therapeutic approach for AMD.

As discussed earlier, gut microbiota affect all aspects of immune development and homeostasis in health and disease [9]. For this reason, the intestinal microbiota represent candidates for the interplay between the genetic and environmental factors that cause AMD. Using mouse models of neovascular AMD, it has been demonstrated that HFDs exacerbated choroidal neovascularization by altering gut microbiota [207]. Gut dysbiosis instead leads to heightened IP and chronic low-grade inflammation, characteristic of inflammaging, with elevated production of IL-6, IL-1β, TNF-α, and VEGF-A. Both antibiotic treatment and microbiotal transplants (T) from chow diet (CD)-fed mice restored microbial proportions, i.e., Bacteroidetes to Firmicutes ratio of 3 to 1. In addition, improved glucose tolerance, lower systemic and choroidal inflammation, and IP in HFDxCDT mice compared to HFDxHFDT mice were observed [207]. Furthermore, mice fed high-glucose diet (HGD) developed a dysbiosis associated with AMD-like features, such as RPE and photoreceptor atrophy, and lipofuscin accumulation [208]. In addition, HGD-fed mice showed lower microbial diversity than CD-fed animals, with a reduced abundance of Bacteroidetes and an increased level of Proteobacteria at the phylus levels (one of the best source of LPS), as well as higher levels of the Desulfovibrio vulgaris species. A significant increase of IP, demonstrated by a reduced expression of tight junction proteins, such as ZO-1 and occludin, was also observed [209]. 

In a clinical study, AMD patients displayed significant gut microbial alterations compared with healthy controls. Moreover, AMD associated gut bacteria were immunologically relevant, enriched in the IgA-bound fraction, and contained altered microbes relating to fatty acid metabolism and carotenoid pathway biosynthesis, compared to controls [210]. A cross-sectional study reported that the western diet, rich in dairy products, red meats, and eggs, was significantly associated to a higher incidence of advanced AMD compared with the oriental diet, rich in vegetable, legumes, rice, fruits, low-fat dairy products and fish [211]. 

#### 4.2.2. Glaucoma

Glaucoma is a term defining a group of optic degenerative diseases characterized by the progressive death of retinal ganglion cells (RGCs) and excavation of the optic nerve head (ONH) [212]. Glaucoma is the leading cause of global irreversible blindness. The number of people with glaucoma worldwide (aged 40 e 80 years) will increase from 64.3 million in 2013 to 111.8 million in 2040, disproportionately affecting people residing in Asia and Africa [213]. Primary open-angle glaucoma is characterized by a particular abnormal appearance of the optic- nerve disk, whereas pigmentary glaucoma is a secondary glaucoma showing: (1) Disruption of the posterior iris-pigment epithelium; (2) dispersion of the pigment throughout the anterior segment and the trabecular mesh work; and (3) increase in intraocular pressure (IOP) [214]. Age, elevated IOP, race, family history, myopia, diabetes, and para-inflammation are involved in the pathogenesis, development and progression of glaucoma (Figure 5) [215] [216,217,218]. Moreover, several data have suggested that the immune system is involved even before the normal signs of glaucoma begin [217,219]. The up to date treatments for the early management of the glaucoma have involved the reduction of IOP, via topical instillation of anti-glaucoma eye drops, laser therapy or invasive surgery [220]. Preclinical and clinical studies have demonstrated the protective effects of n-3 PUFAs in different retinal disease [221,222,223], including glaucoma [224]. Interestingly, EPA and DHA plasma levels found in patients affected by primary open-angle glaucoma were lower than those measured in healthy subjects [225]. In addition, in an experimental model of hereditary glaucoma [226], the administration of n-3 PUFAs, alone or in combination with timolol, displayed neuroprotective effects only when the ratio of AA to Epa was kept between 1 and 1.5 [227]. This result was associated with the downregulation of IL-18 and TNF-α expression only when n-3 PUFAs were administrated alone, which indicated that the neuroprotection in the retina might be mediated by other mechanisms, i.e., the synthesis of SPMs were able, not only to resolve inflammation, but also protected organs and stimulate tissue regeneration (Figure 2) [228]. It has been demonstrated that RGCs are the most susceptible retinal neurons to high IOP [229]. Ocular hypertension also lead to an alteration of visual function [230], since both a and b-wave amplitudes of the electroretinogram (ERG) were decreased after a marked elevation of IOP caused by an ischemia-reperfusion sequence [231,232]. In an in vivo rat model of IOP elevation, it has been demonstrated that dietary supplementation of n-3 and n-6 PUFAs was able to reduce retinal stress and preserve retinal structure [233]. In line with these data, Bazan and co-workers demonstrated that the oxygenated metabolite of DHA, neuroprotectin D1 (NPD1), was responsible of these neuroprotective effects (Figure 2) [234,235]. NPD1 was shown to decrease: (i) The production of inflammatory molecules (such as COX-2, IL-1β); (ii) to up-regulate anti-apoptotic Bcl-2 proteins; and (iii) to down-regulate pro-apoptotic proteins (Bax, Bad, Caspase-3) [233,236].

Recently, in a mouse model, it was shown that a transient elevation of IOP was sufficient to induce T-cell infiltration into the retina and this step was essential for the development of glaucoma. In addition, the authors demonstrated that T-cell activations and glaucomatous neurodegeneration were abolished in GF mice, strengthening the hypothesis that a bacteria-sensitized T-cell responses underly the pathogenesis of glaucoma [237]. In line with these data, long-term intake of Lactobacillus paracasei KW3110 has been associated with a more abundance of beneficial gut bacteria and a reduction of the age-related immune dysfunctions, i.e., lowered expansion of pro-inflammatory T cells and serum cytokines [238]. Instead, this probiotic treatment determined a reduction of the Firmicutes to Bacteroidetes ratio, demonstrated by increased Bifidobacteriaceae and decreased Streptococcaceae families. It is important to highlight that Bifidobacterium is known as one of the most beneficial bacterial family [67], and Streptococcaceae bacteria are able to stimulate the intestinal cells to secrete pro-inflammatory cytokines [239].

## 5. Conclusions

Many observational studies have shown the potential role of nutraceuticals and functional foods, such as n3-PUFAs, probiotics and prebiotics, in preventing chronic inflammatory disease or to aid in their treatment. Since IBD, AMD and glaucoma are illnesses increasing at alarming rates, more basic and clinical studies are needed to definitely determine if dietary factors (alone or in combination) are able to prevent the development of these disabling pathologies. The discovery of n-3 PUFAs-derived anti-inflammatory molecules, i.e., SPMs, may offer a fascinating new complementary option for the treatment of these inflammatory-based diseases. Indeed, a new area of research is focusing on the development of synthetic analogs of the natural SPMs as well as on the combination between n-3 PUFAs with drugs acting as regulators of endogenous enzyme activities [240]. The former strategy has been pursued by the OMEICOS Therapeutics and the compound OMT-28 entered clinical phase I in February 2017 (NTC 03906799).

On the other hand, n-3 PUFAs can also be considered prebiotics due to the fact that they are able to increase the production of anti-inflammatory molecules, such as SCFAs. Coherent with this data, recent reports suggested that the combination between prebiotics and probiotics, called symbiotic, bear great potential for correcting the gut dysbiosis and selectively stimulated the growth and/or activated the metabolism of one or a limited number of health-promoting bacteria, aimed at restoring the mutualism of holobiont [241]. Moreover, a better understanding of the mechanisms that underlie microbial resilience towards external perturbations will be a key requirement for microbiome-directed precision medicine [242].

In summary, modulation of the microbiota remains a promising therapeutic option for the prevention and treatment of complex diseases, but much more must be learned to maximize treatment success.
